# Sea Cucumber Body Vesicular Syndrome Is Driven by the Pond Water Microbiome via an Altered Gut Microbiota

**DOI:** 10.1128/msystems.01357-21

**Published:** 2022-04-14

**Authors:** Zelong Zhao, Jingwei Jiang, Yongjia Pan, Ying Dong, Bai Wang, Shan Gao, Zhong Chen, Xiaoyan Guan, Xuda Wang, Zunchun Zhou

**Affiliations:** a Liaoning Key Lab of Germplasm Improvement and Fine Seed Breeding of Marine Aquatic Animals, Liaoning Ocean and Fisheries Science Research Institute, Dalian, Liaoning, People’s Republic of China; University of California Merced

**Keywords:** sea cucumber, *Apostichopus japonicus*, body vesicular syndrome, multiomics, fatty acid metabolism disorder, gut microbiota, vitamin B5

## Abstract

Apostichopus japonicus (sea cucumber) is one of the most valuable aquaculture species in China; however, different diseases can limit its economic development. Recently, a novel disease, body vesicular syndrome (BVS), was observed in *A. japonicus* aquaculture. Diseased animals displayed no obvious phenotypic characteristics; however, after boiling at the postharvest stage, blisters, lysis, and body ruptures appeared. In this study, a multiomics strategy incorporating analysis of the gut microbiota, the pond microbiome, and *A. japonicus* genotype was established to investigate BVS. Detailed analyses of differentially expressed proteins (DEPs) and metabolites suggested that changes in cell adhesion structures, caused by disordered fatty acid β-oxidation mediated by vitamin B5 deficiency, could be a putative BVS mechanism. Furthermore, intestinal dysbacteriosis due to microbiome variations in pond water was considered a potential reason for vitamin B5 deficiency. Our BVS index, based on biomarkers identified from the *A. japonicus* gut microbiota, was a useful tool for BVS diagnosis. Finally, vitamin B5 supplementation was successfully used to treat BVS, suggesting an association with BVS etiology.

**IMPORTANCE** Body vesicular syndrome (BVS) is a novel disease in sea cucumber aquaculture. As no phenotypic features are visible, BVS is difficult to confirm during aquaculture and postharvest activities, until animals are boiled. Therefore, BVS could lead to severe economic losses compared with other diseases in sea cucumber aquaculture. In this study, for the first time, we systematically investigated BVS pathogenesis and proposed an effective treatment for the condition. Moreover, based on the gut microbiota, we established a noninvasive diagnostic method for BVS in sea cucumber.

## INTRODUCTION

**FIG 1:**
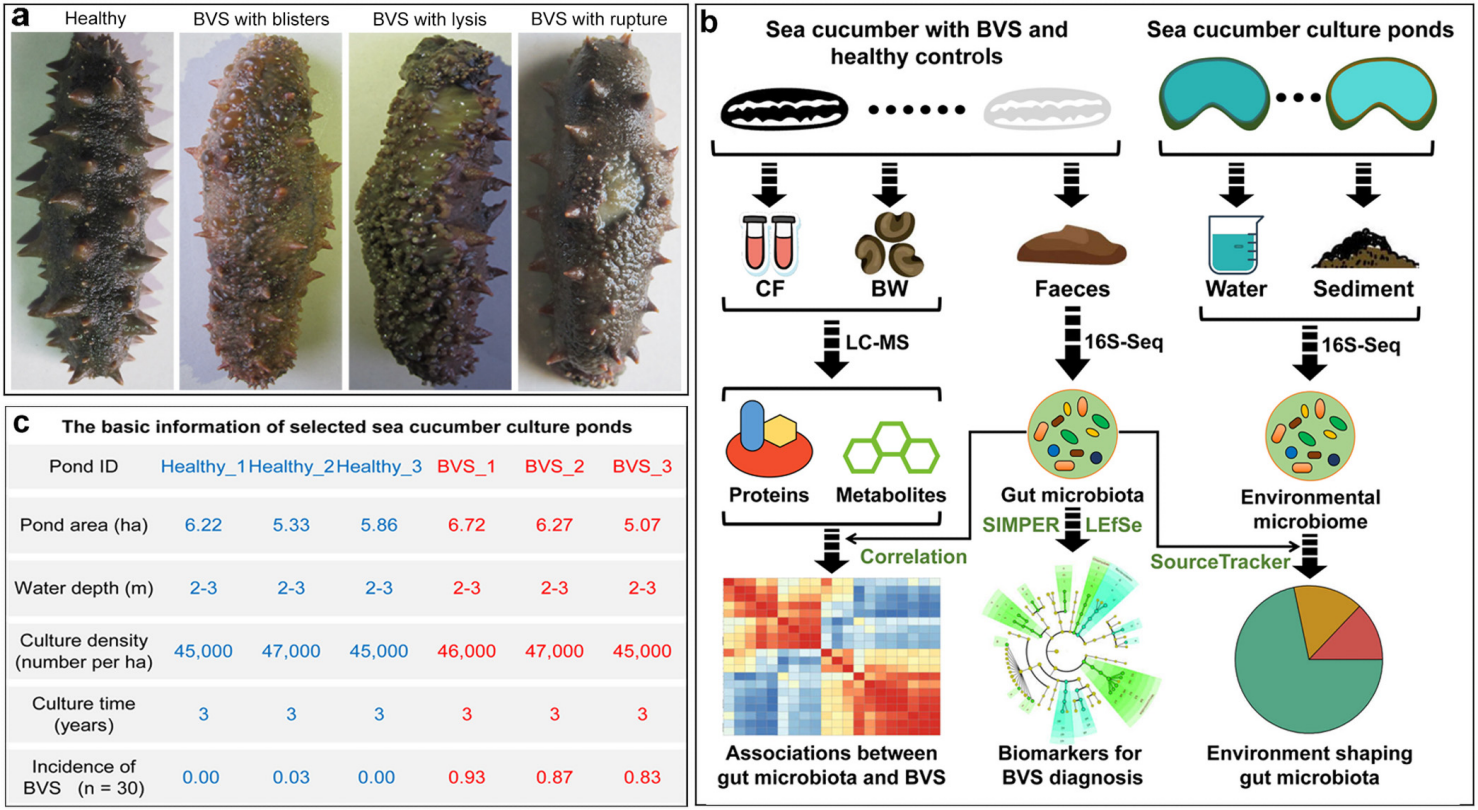
Study information. (a) Morphological differences in healthy and BVS *A. japonicus* animals after boiling. (b) Multiomics study framework showing the investigation of BVS molecular mechanisms and the identification of biomarkers related to BVS diagnosis. (c) Basic information on *A. japonicus* culture ponds in this study.

The sea cucumber (Apostichopus japonicus) is one of the most dominant and economically important species on northeastern Asia coasts (1) due to its considerable nutritive and medicinal value (2). With an increasing market demand for such products, the sea cucumber aquaculture industry has rapidly developed in recent years (3). However, frequent disease outbreaks in *A. japonicus* aquaculture have severely restricted the development of this industry and caused huge economic losses (4). The most common *A. japonicus* disease, skin ulceration syndrome (SUS), manifests several symptoms, including skin ulceration, evisceration, general atrophy, swollen mouth, and anorexia (5), and leads to 90% to 100% mortality (6). In recent decades, several studies successfully investigated SUS occurrence and therapy in *A. japonicus* aquaculture, leading to effective control of disease incidence (7–9). However, a novel *A. japonicus* disease, body vesicular syndrome (BVS), was recently identified in Liaoning Province (10), one of the highest-producing *A. japonicus* areas in China (11). Distinct from SUS, BVS has no obvious external phenotypic characteristics and is not fatal to *A. japonicus*. The major characteristics are noted after boiling, e.g., blisters, lysis, and rupture (Fig. 1a); however, boiling is the main sea cucumber cooking process, and therefore, these characteristics render the sea cucumber inedible (10). BVS is also very difficult to confirm during aquaculture, and becomes evident only postharvest, upon boiling. This means that sea cucumbers cannot be sold when BVS is detected. To address this situation, early warning BVS signals must be recognized and underlying BVS molecular mechanisms elucidated to prevent and treat this disease during *A. japonicus* aquaculture.

In recent years, the rapid development of high-resolution mass spectrometry (MS) has revolutionized the systematic investigation of molecular responses in aquaculture species with different diseases (12–14). In *A. japonicus*, proteomics showed that α-5-collagen and ATP5-β have important roles during SUS progression (15). Similarly, nuclear magnetic resonance (NMR)-based metabonomics identified enhanced energy storage and immune responses in *A. japonicus* with SUS (16). In addition to host-mediated molecular responses, gut microbiota-host interactions have become highly topical in studying host diseases (17–19). In humans, gut microbiota dysbiosis is associated with diverse diseases, including type 2 diabetes (15), inflammatory bowel disease (20), polycystic ovary syndrome (21), and obesity (22). Moreover, significant gut microbiota variations have been identified in many diseased aquaculture species compared with healthy counterparts, including diverse fish species (23), Pacific white shrimp (Litopenaeus vannamei) with white feces syndrome (24), and *A. japonicus* with SUS (15). Due to its lack of external disease characteristics and its nonlethality, we speculate that BVS is not caused by a specific pathogen, as for SUS (25), but is more likely a physiological and metabolic disease caused by gut microbiota dysbiosis (26).

Recently, several studies reported that differences in gut microbiota in hosts were related to the host’s environment (27, 28). Indeed, *A. japonicus* has several features which make it ideal for investigating how living environments shape gut microbiotas. First, *A. japonicus* is a detritus feeder and processes huge volumes of water and sediment through the intestinal tract (29); therefore, environmental bacterial communities possibly shape the gut microbiota (30). Second, *A. japonicus* enters a dormant state (aestivation) to protect itself at high temperatures. In this state, animals do not eat, and their intestines gradually degenerate (31). When dormancy ends, the intestinal tract regenerates and the gut microbiota develops anew (32). Third, *A. japonicus* has a unique defensive mechanism (evisceration) (33). When stressed or threatened, animals evacuate their internal organs from the body, and after a few days, missing organs are regenerated (34). These aestivation and visceral regeneration processes allow microbes in surrounding environments to colonize the new intestinal tract and form an unfamiliar homeostasis (35). Therefore, by investigating how aquaculture environments contribute to *A. japonicus* gut microbiota, coupled with host health status, we can expand our understanding of the gut microbiota during BVS occurrence and identify associated mechanisms.

We used a multiomics strategy to investigate molecular response mechanisms of BVS and associations between the health state and gut microbiota in *A. japonicus* (Fig. 1b). In diseased animals, we observed a loose body wall structure and disordered fatty acid metabolism, which suggested vitamin B5 deficiency mediated by gut dysbiosis. Several disease-discriminating biomarkers were identified in the gut microbiota and were useful for diagnosing BVS. Moreover, we discovered a significant influence of the culture pond microbiome (water fraction) on *A. japonicus* gut microbiota and health status. Finally, a vitamin B5 supplementation study was performed to verify the putative B5 deficiency mechanism and treat BVS in *A. japonicus*.

## RESULTS AND DISCUSSION

We used an integrated experimental and bioinformatics strategy to identify interrelationships between BVS phenotypes, gut microbiota, and the environmental microbiome in an *A. japonicus* aquaculture ecosystem (Fig. 1b). To characterize proteomic and metabolic profiles, and gut microbiome composition in BVS animals, we collected and analyzed body wall (BW), coelomic fluid (CF), and stool samples from 11 *A. japonicus* individuals with BVS and 11 non-BVS healthy controls. These individuals were representative of BVS and healthy *A. japonicus* animals in six experimental aquaculture ponds (four individuals from two ponds and three from another pond). The characteristics and incidence of BVS in selected culture ponds are summarized (Fig. 1c). BW and CF samples from individuals in the same ponds were mixed and measured using liquid chromatography-mass spectrometry (LC-MS) to evaluate expressed proteins and metabolites. Stool samples were subjected to 16S rRNA amplicon sequencing, followed by microbial community taxonomic composition profiling. Additionally, water and sediment samples from each *A. japonicus* pond were analyzed using 16S rRNA amplicon sequencing, and contributions from the environmental microbiome to host gut microbiota were measured using SourceTracker (36).

### BVS alters proteomic and metabolic profiles in *A. japonicas*.

**FIG 2:**
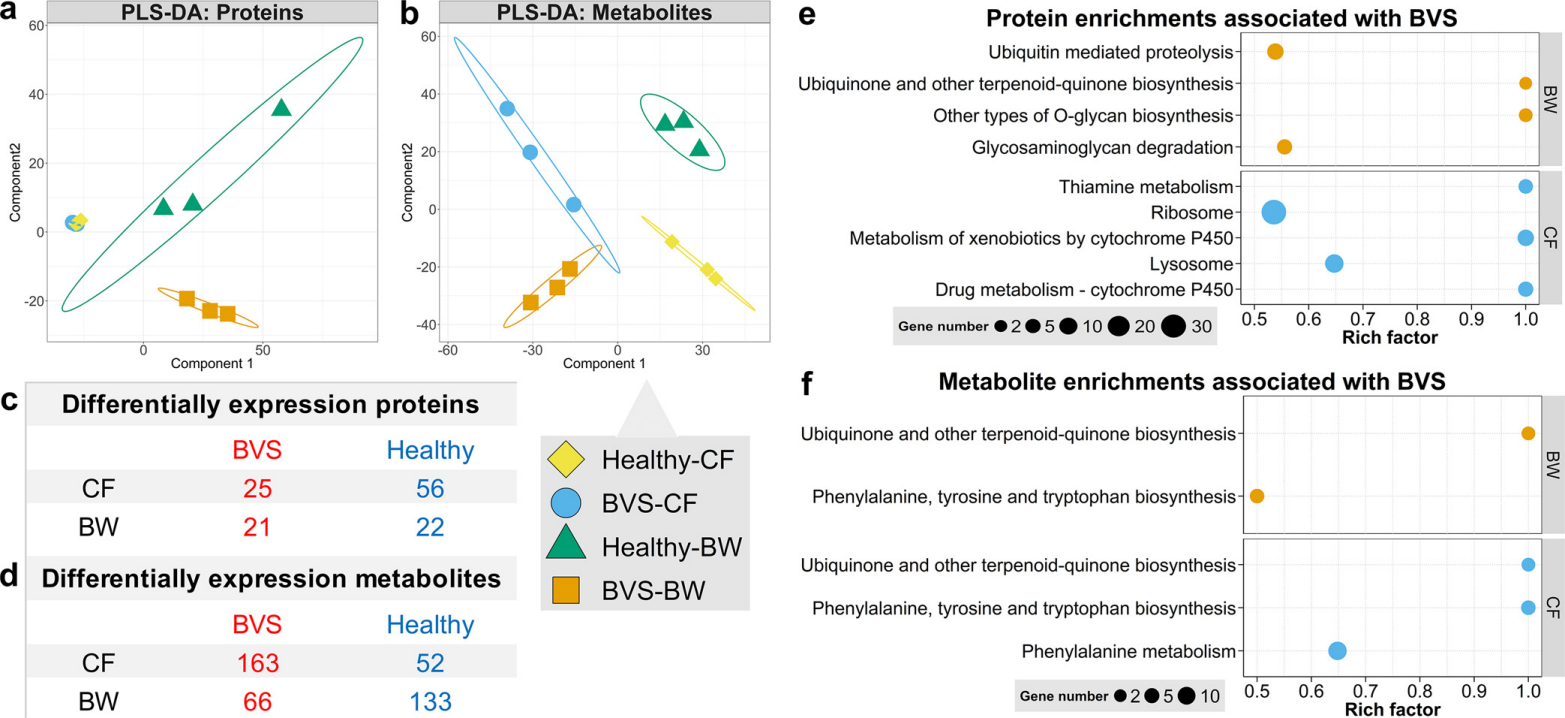
Proteomic and metabolic response profiles for BVS in *A. japonicus*. PLS-DA identified changes in proteomic (a) and metabolic (b) profiles in BW and CF samples between healthy and BVS animals. Points with different shapes and colors denote different samples. The numbers of DEPs (c) and DAMs (d) in BW and CF samples between healthy and BVS animals. Red and blue numbers correspond to proteins and metabolites upregulated in BVS and healthy animals. KEGG enrichment analysis based on DEPs (e) and DAMs (f) in BW and CF samples between healthy and BVS animals. The dot size denotes the numbers of genes in each pathway related to DEPs or DAMs in different samples. Only data with adjusted *P* values of <0.05 are shown.

To explore BVS etiology in *A. japonicus*, an LC-MS-based proteomics and metabolomics strategy was used to investigate protein and metabolite variations in CF and BW samples. In total, >2,000 proteins and >4,000 metabolites were identified based on tandem mass spectrum and ion current profiles, respectively. Partial least-squares discriminant analysis (PLS-DA) identified significant cluster separation between healthy and BVS groups with respect to CF and BW samples, except for CF protein patterns (Fig. 2a and b). Protein expression comparisons identified 81 and 43 differentially expressed proteins (DEPs) in CF and BW samples, respectively (fold change > 1.5; adjusted *P* value < 0.05) (Fig. 2c). Also, 215 and 199 metabolites, at significantly different levels, were identified in CF and BW samples, respectively (variable importance in projection [VIP] value > 1; fold change > 2; adjusted *P* value < 0.05) (Fig. 2d). These expression differences indicated broad metabolic regulatory shifts between healthy and diseased animals.

Proteome maps showed that DEPs in CF were significantly enriched in ribosomes, lysosomes, thiamine metabolism, and cytochrome P450-related metabolic pathways (pathway impact value > 0.2; adjusted *P* value < 0.05) (Fig. 2e). These pathways were involved in vitamin metabolism and the stabilization of normal physiological functions in hosts (37–39). In contrast, DEPs in BW samples were significantly enriched in *O*-glycan biosynthesis, glycosaminoglycan degradation, ubiquitin mediated proteolysis, and ubiquinone-related metabolism pathways (pathway impact value > 0.2; adjusted *P* value < 0.05) (Fig. 2e). Glycans have multiple functions in organisms: (i) as signaling molecules in protein folding and lysosome degradation (40), (ii) as cell membrane structural components in synovial and soft tissues (41), and (iii) as pattern recognition receptors associated with cell adhesion in tissue formation (42) and immune responses (43). Ubiquitin is a small regulatory protein involved in ubiquitination; the process marks different proteins for degradation via the proteasome, alters cellular locations, and affects activity (44). Our enrichment analysis using DEPs identified disordered vitamin metabolism and protein structures in CF and BW samples, respectively, in BVS animals.

Moreover, metabolome enrichment analysis was also performed, with differentially abundant metabolites (DAMs) significantly enriched in ubiquinone- and phenylalanine-related metabolism pathways in both CF and BW samples (pathway impact value > 0.2; adjusted *P* value < 0.05) (Fig. 2f). Ubiquinones are electron carriers which function during oxidative phosphorylation and are required for energy production (45). The quinoid nucleus of ubiquinone is derived from either chorismate or tyrosine via phenylalanine metabolism (46). These findings suggested that BVS alters energy metabolism levels in *A. japonicus*.

### Putative mechanisms underpinning the BVS phenotype.

**FIG 3:**
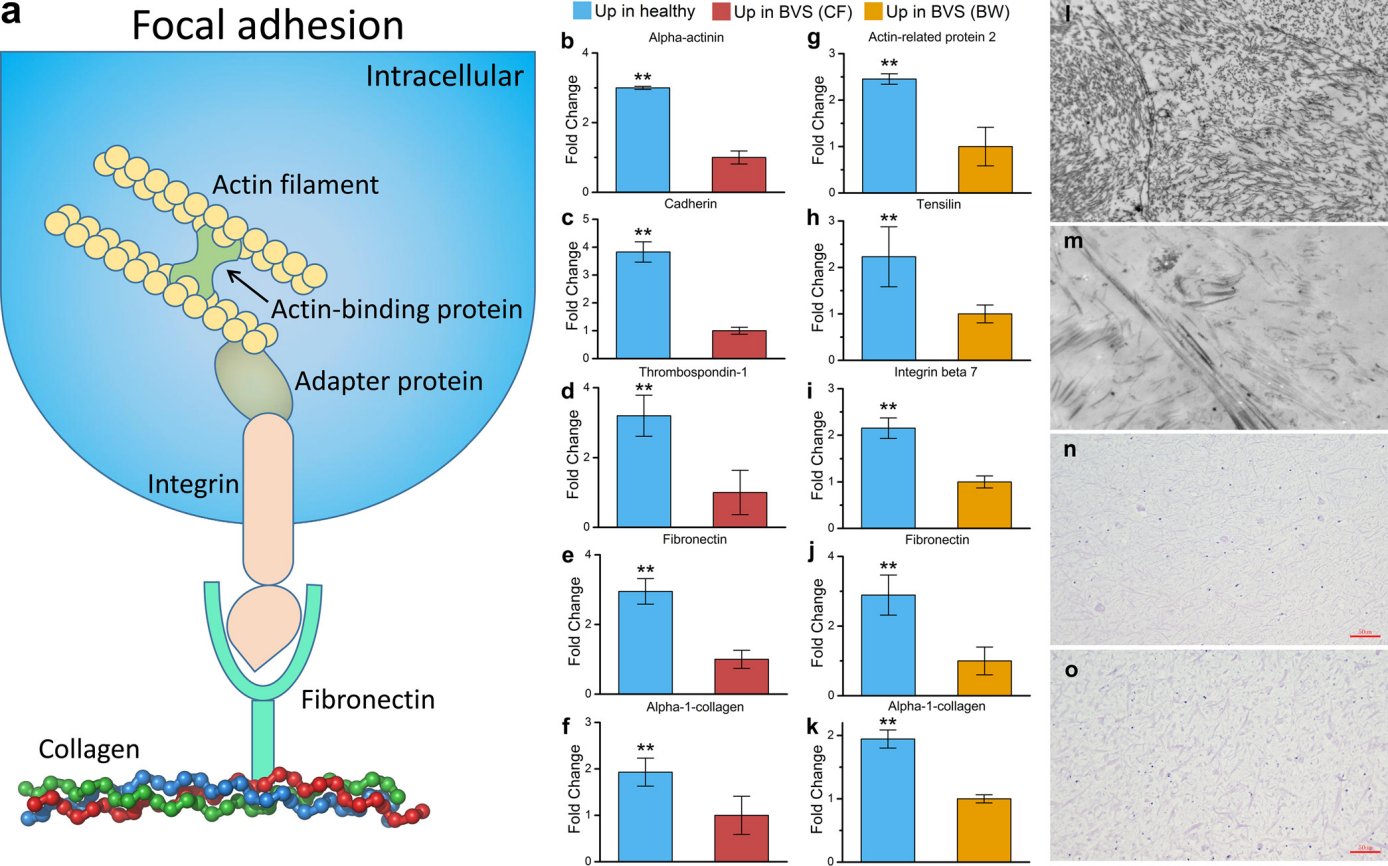
Focal adhesion processes are associated with BVS morphological characteristics. (a) Conceptual framework of focal adhesion processes and related proteins. (b to f) Proteins associated with different components of focal adhesion processes downregulated in the CF of BVS animals compared with healthy animals. (g to k) Proteins associated with different components of focal adhesion processes downregulated in the BW of BVS animals compared with healthy animals. **, *P* < 0.05. (l and m) BW of healthy and BVS animals under high-resolution microscopy (bar = 2 μm). (n and o) BW of healthy and BVS animals under light microscopy after H&E staining (bar = 50 μm).

Our DEP data suggested that focal adhesion subcellular structures, required for tissue formation, had deteriorated and potentially caused the BVS phenotype in *A. japonicus* (Fig. 3). Focal adhesion is characterized by large, dynamic protein complexes, through which the cytoskeleton connects to the extracellular matrix (47). The cytoskeleton is composed of actin filaments which function via multiple actin-binding proteins (48). The intracellular domain of integrin binds to the cytoskeleton via adapter proteins such as filamin, vinculin, and tensin (49). In turn, these integrins bind to extracellular proteins, such as fibronectin and collagen, via short amino acid sequences (50). The skeleton of focal adhesion related to the BVS was inferred based on the results of DEPs in CF and BW (Fig. 3a).

Focal adhesion proteins associated with muscle formation were consistently downregulated in the CF from BVS animals compared with healthy animals (Fig. 3b to f). Actin filaments are linked to α-actinin and to membranes via cadherin, which were 3.00- and 3.83-fold downregulated in the CF of BVS animals, respectively (Fig. 3b and c). The α-actinin is required for the attachment of actin filaments to Z lines in skeletal muscle cells and to dense bodies in smooth muscle cells (51). Cadherins have vital roles in cell migration through the epithelial-mesenchymal transition process (52), which is required to maintain muscle structures in *A. japonicus* (53). The integrin thrombospondin-1 was 3.19-fold downregulated in the CF of BVS animals (Fig. 3d) and was shown to modulate endothelial cell adhesion and bind fibronectin (54). Last, the extracellular matrix, including fibronectin and α-1-collagen, was 2.95- and 1.93-fold downregulated in the CF of BVS animals (Fig. 3e and f). In the CF, integrin-bound-fibronectin readily interacts with collagen, which is a major component of endomysium in muscle, which assembles the soluble fibronectin into insoluble form to separate the muscle tissue (55).

Similar results were identified for DEPs in BW samples; however, the focal adhesion complex in BW displayed components different from those seen in CF analysis (Fig. 3g to k). The extracellular matrix in BW focal adhesions was composed of fibronectin and α-1-collagen, which were 2.89- and 1.94-fold downregulated in BVS animals (Fig. 3j and k). The integrin protein integrin β-7 was 2.15-fold downregulated in these animals (Fig. 3i). Also, the actin-binding and adapter proteins, actin-related protein 2 and tensilin, were 2.45- and 2.23-fold downregulated in animals (Fig. 3g and h). In nonmuscle cells, the filament nucleator actin-related protein 2/3 complex binds to the side of actin filaments to generate branched networks (56). Actin filaments then bind tensilin, which contains a phosphotyrosine-binding domain at the C terminus which interacts with the cytoplasmic tail of β-integrin (57).

Focal adhesion destruction in the muscle and BW of *A. japonicus* with BVS was confirmed by histology (Fig. 3l to o). First, *A. japonicus* BW morphology was observed using ultrahigh-resolution microscopy. Healthy BW exhibited compact fibrous structures (Fig. 3l), while diseased animals had incomplete morphological structures (Fig. 3m). Disordered BW structures in diseased animals were also identified using hematoxylin-and-eosin (H&E) staining under light microscopy. These structures were coarser and contained looser fiber structures than those in healthy animals (Fig. 3n and o). When these observations were combined, it was hypothesized that focal adhesion disruption is a primary cause of the BVS phenotype.

### BVS-linked metabolite associations with host physiological functions.

**FIG 4:**
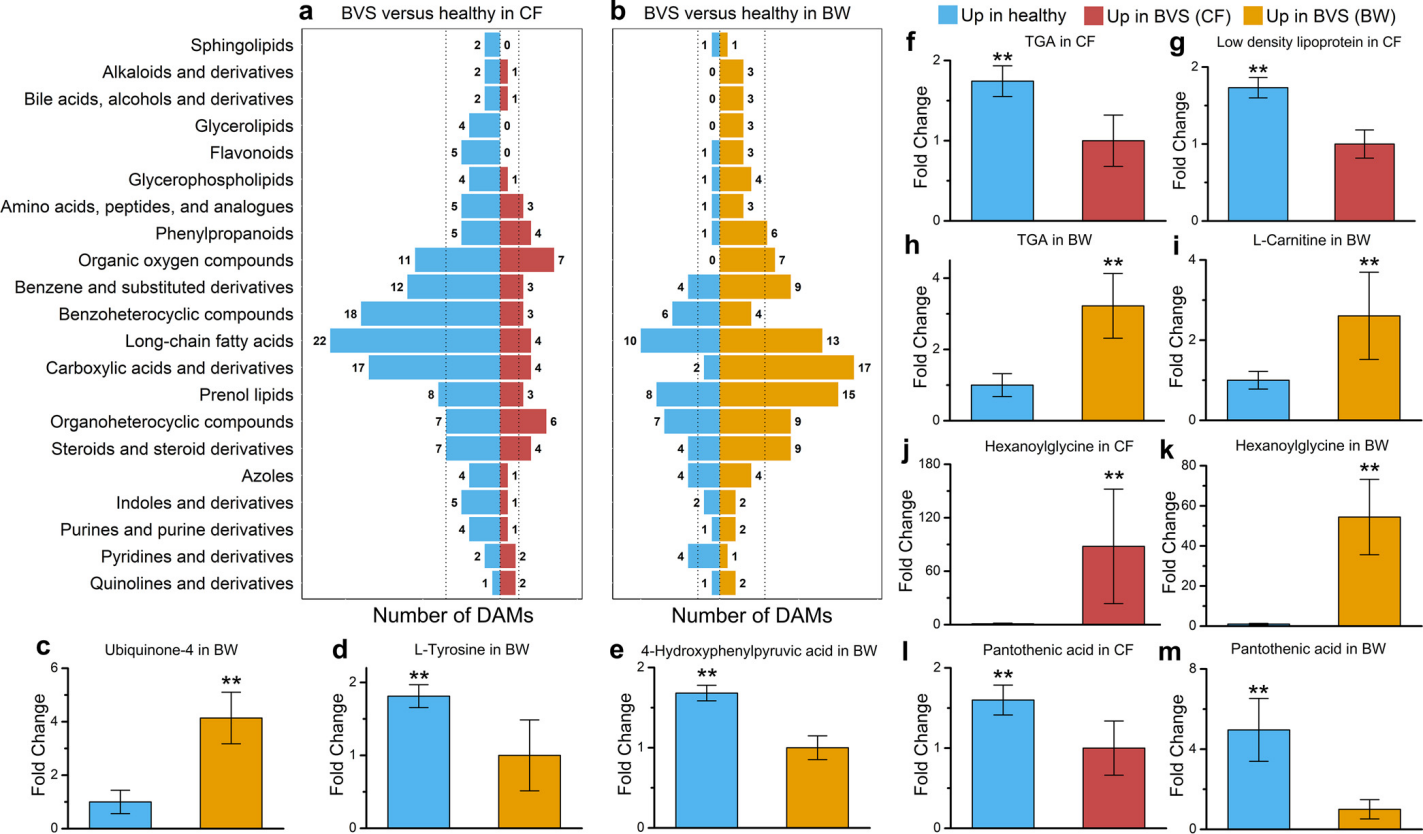
The metabolomic identification of disordered fatty acid metabolism in BVS *A. japonicus*. Different DAM classes identified in CF (a) and BW (b) samples between healthy and BVS *A. japonicus*. (c to e) Metabolites associated with energy production that were downregulated in the BW of BVS *A. japonicus* compared with healthy animals. (f to k) Metabolites associated with disordered fatty acid metabolism that were differentially expressed in the CF and BW of BVS *A. japonicus* compared with healthy animals. (l and m) Variations in vitamin B5 levels in CF and BW samples from healthy and BVS *A. japonicus*. **, *P* < 0.05.

To comprehensively dissect metabolic changes in BVS, we defined metabolite classes based on Human Metabolome Database (HMDB) annotation and focused on 21 metabolite classes with at least four DAMs in the data set. Most metabolite classes were significantly depleted in CF-diseased animals compared with healthy animals (Fig. 4a). In contrast, more metabolite classes were significantly enriched in the BW of BVS animals compared with healthy individuals (Fig. 4b). Specifically, diverse flavonoids and phenylpropanoids, with significant abundance differences, were identified as phenylalanine metabolism intermediates and derivatives (Fig. 4c to e). Ubiquinone 4 (coenzyme Q) was more abundant in the BW of diseased animals (Fig. 4c), suggesting increased energy synthesis (45). Also, the depleted quinoid nucleus synthesis substrates tyrosine and 4-hydroxyphenylpyruvic acid were identified in the BW of diseased animals (Fig. 4d and e) and showed that ubiquinone in *A. japonicus* was created from phenylalanine and mediated by the tyrosine pathway (46). These results, coupled with enriched DAM pathways (Fig. 2f), indicated an important role for phenylalanine in response to BVS.

The greatest metabolite change in our data set was to long-chain fatty acids (Fig. 2a and b). Metabolites belonging to prenol lipid, steroid, and glycerophospholipid classes were also differentially abundant in CF and BW samples from diseased animals relative to healthy individuals (Fig. 2a and b). These changes perturbed fatty acid metabolism in these animals. Triacylglycerol (TAG) is an important and common glycerophospholipid and was depleted in the CF of diseased animals (Fig. 4f), suggesting too few available lipids for normal metabolism. We also observed downregulated low-density lipoprotein in CF samples from diseased animals (Fig. 4g), suggesting that lipid transport may be inhibited during BVS (58). TAG abundance in the BW was much lower than in the CF in the same individual, but significant TAG accumulation was observed in the BW of *A. japonicus* with BVS (Fig. 4h). Also, l-carnitine was enriched in the BW of diseased animals compared with healthy individuals (Fig. 4i); l-carnitine binds to long-chain fatty acids and transports them into the mitochondria to generate energy via oxidation (59). The l-carnitine-mediated entry process is a rate-limiting factor and an important point of regulation for fatty acid β-oxidation (60). Also, our metabolomics strategy highlighted hexanoylglycine, a fatty acid β-oxidation disorder biomarker (61), as the metabolite with the greatest abundance change in both CF and BW samples from diseased animals (54.42- and 87.89-fold increases, respectively) (Fig. 4j and k). l-Carnitine binding to fatty acids requires coenzyme A, which is produced via pantothenic acid (vitamin B5) metabolism (62). Significantly depleted pantothenic acid levels were identified in both CF and BW samples from diseased animals (Fig. 4l and m). Thus, vitamin B5 deficiency could underlie the BVS epidemic in *A. japonicus*, where disordered fatty acid β-oxidation occurs via inhibited l-carnitine-fatty acid binding.

### Associations between gut microbiota and host metabolism in BVS.

**FIG 5:**
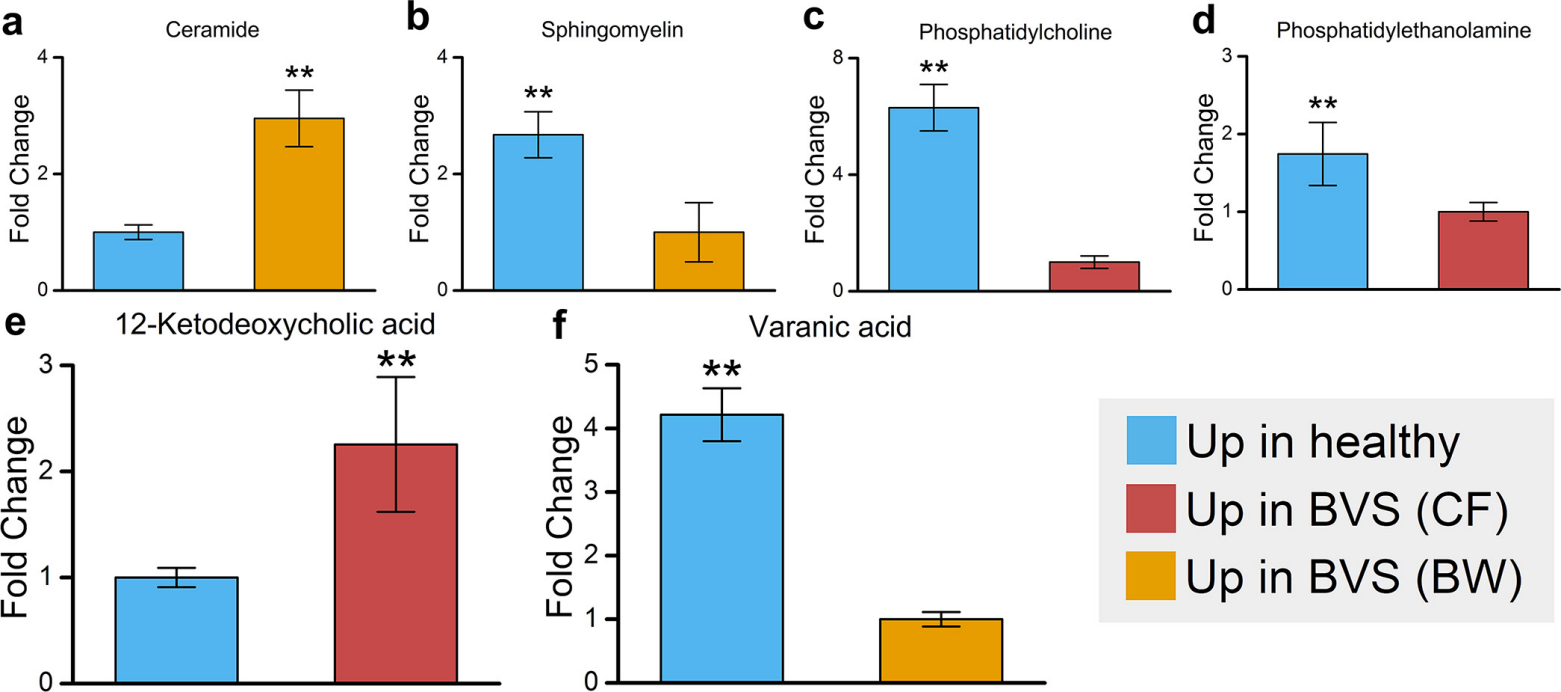
Associations between gut microbiota and host metabolism in *A. japonicus* with BVS. (a to d) Metabolites associated with disordered sphingolipid metabolism that were differentially expressed in CF and BW of BVS *A. japonicus* compared with healthy animals. (e and f) Metabolites associated with disordered bile acid metabolism that were differentially expressed in the CF and BW of BVS *A. japonicus* compared with healthy animals. **, *P* < 0.05.

Secondary metabolites produced by the gut microbiota can significantly affect host metabolism (63). Sphingolipids are an important metabolite class associated with the gut microbiota; they are prevalent in *Bacteroidetes* membranes and also modulate the inflammation state of the host (64). Two compounds were identified as DAMs in our data set: ceramide was overabundant in the BW of diseased animals, while sphingomyelin was overabundant in the BW of healthy *A. japonicus* (Fig. 5a and b). Sphingomyelin is a major component in the lipid bilayer of cell membranes and is composed of ceramide and phosphocholine (65). We also detected a significant depletion of phosphocholine and its synthetic precursor, phosphatidylethanolamine, in the CF of diseased animals compared with healthy controls (Fig. 5c and d).

Bile acids are another metabolite class associated with the gut microbiota, with multiple roles in host metabolism (66). Primary bile acids are secreted by host tissue and deconjugated by microbes to secondary bile acids in the healthy gut where they promote lipid digestion (67). The secondary bile acid, 12-ketodeoxycholic acid, was depleted in the CF of diseased animals (Fig. 5e), while the complementary enrichment of the primary bile acid precursor, varanic acid, was observed in the BW of diseased animals (Fig. 5f). These findings suggested that disordered fatty acid metabolism in diseased animals was putatively linked with weakened sphingolipid and bile acid metabolism in the gut microbiota.

### Gut microbiota signatures differentiate BVS.

The BVS phenotype was identifiable only after boiling; therefore, it was critical to develop a live-animal assay to detect BVS during *A. japonicus* culture. Disease detection methods based on gut microbiota biomarkers have been widely used in clinical practice (20) and, importantly, also applied to aquaculture activities (68). To determine if gut microbiota differences could be used to classify diseased *A. japonicus* according to BVS phenotype, gut microbiota composition was analyzed. *Rhodobacterales* was the most dominant bacterial order in the gut microbiota of both BVS and healthy *A. japonicus*; however, it was more abundant in BVS individuals (Fig. S1). Additionally, the relative abundance of *Cellvibrionales*, *Chloroplast*, *Desulfobulbales*, *Bacillales*, and *Campylobacterales* was also significantly higher in the gut microbiota of BVS *A. japonicus* than that of healthy individuals (Wilcoxon test, *P* value < 0.05) (Fig. S1). In contrast, the relative abundance of *Cytophagales* and *Desulfovibrionales* was significantly higher in the gut microbiota of healthy *A. japonicus* than that of diseased individuals (Wilcoxon test, *P* value < 0.05) (Fig. S1). Moreover, distinct variation patterns between BVS and healthy phenotypes were apparent in microbial composition profiles from *A. japonicus* gut microbiota (*P* value < 0.01, permutational multivariate analysis of variance [PERMANOVA]) (Fig. S2).

10.1128/msystems.01357-21.1 FIG S1 Differences in relative abundances of dominant orders detected in gut microbiota between the healthy and BVS *A. japonicus*. *, *P* < 0.05; **, *P* < 0.01; ***, *P* < 0.001. Download FIG S1, JPG file, 0.05 MB. Copyright © 2022 Zhao et al. 2022 Zhao et al. https://creativecommons.org/licenses/by/4.0/ This content is distributed under the terms of the Creative Commons Attribution 4.0 International license.

10.1128/msystems.01357-21.2 FIG S2 PCoA for gut microbiotas of *A. japonicus* based on composition structures. Download FIG S2, JPG file, 0.2 MB. Copyright © 2022 Zhao et al. 2022 Zhao et al. https://creativecommons.org/licenses/by/4.0/ This content is distributed under the terms of the Creative Commons Attribution 4.0 International license.

**FIG 6:**
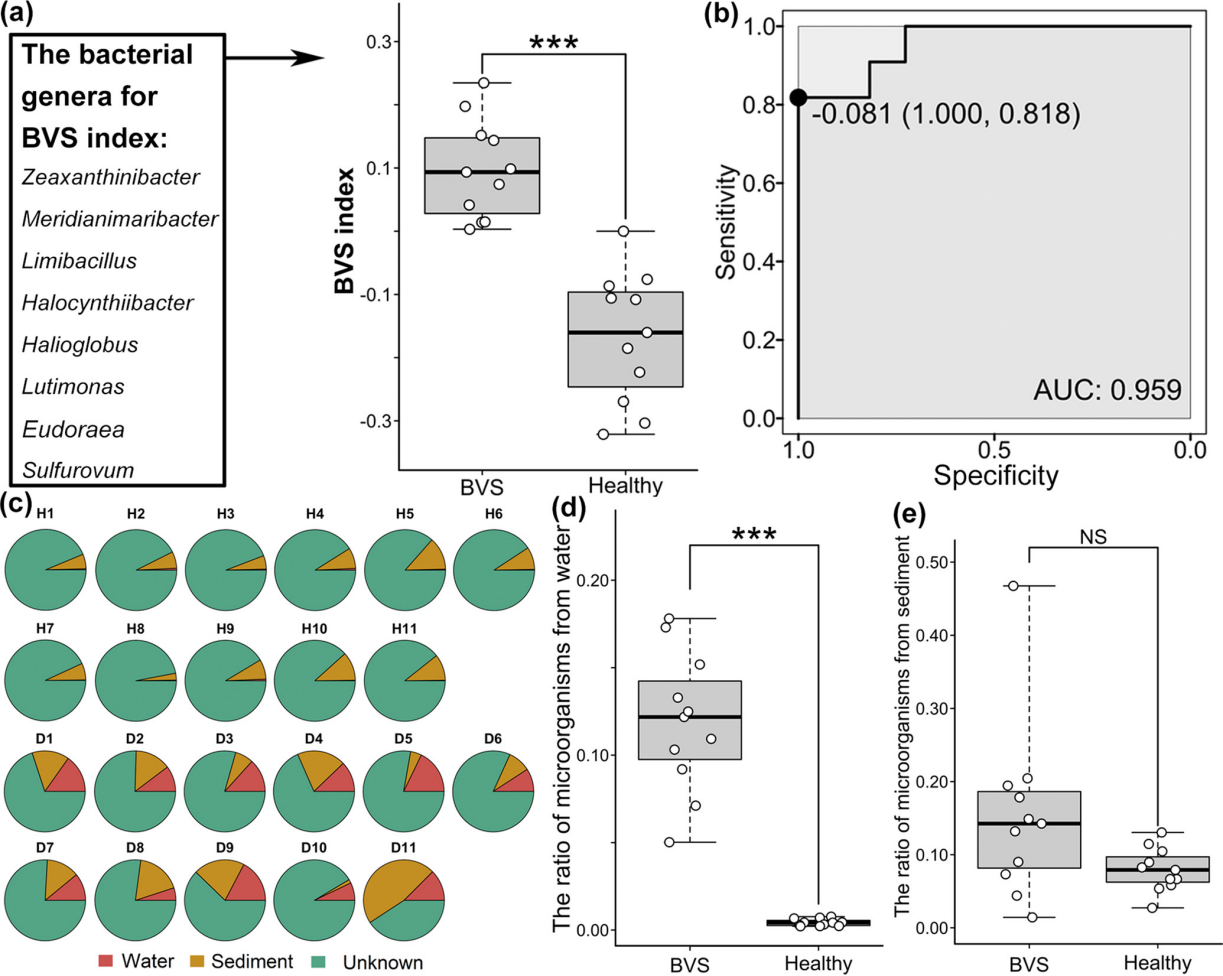
Associations between microbial communities and BVS in *A. japonicus*. (a) Biomarkers identifying gut microbiota and the disease index for BVS diagnosis. (b) ROC curves evaluating accuracy, susceptibility, and diagnosis thresholds for *A. japonicus* with BVS. (c) Ratio of gut microbiotas sourced from water and sediments in culture ponds. (d) Ratio differences in water-sourced microbes between the gut microbiotas in BVS and healthy *A. japonicus*. (e) Ratio differences in sediment-sourced microbes between the gut microbiotas of BVS and healthy *A. japonicus*. ***, *P* < 0.01; NS, not significant.

We used linear discriminant analysis effect size (LEfSe) (69) to identify differential genera by comparing BVS animals with healthy controls. In total, 15 genera were identified as significantly increased or decreased in BVS animals (LDA > 2, *P* value < 0.01) (Fig. S3). We then applied a method based on SIMPER to further screen biomarkers from these 15 genera (70). Finally, eight genera were recognized, and of these, four (*Zeaxanthinibacter*, *Meridianimaribacter*, *Limibacillus*, and *Halocynthiibacter*) were enriched in diseased animals, while the others (*Halioglobus*, *Lutimonas*, *Eudoraea*, and *Sulfurovum*) were enriched in healthy controls (Fig. 6a). Based on these eight biomarkers, a BVS index, indicating differences in the relative abundance of biomarkers enriched in BVS and healthy organisms, was established to measure overall gut microbiota shift severity in diseased animals with respect to healthy controls. Critically, the BVS group had significantly lower BVS index values (*P* value < 0.01, Wilcoxon test) (Fig. 6a). We also generated receiver operating characteristic (ROC) curves to diagnose diseased animals based on BVS index values; the optimal BVS index threshold was chosen at the ROC cutoff point, maximized using Youden’s *J* statistic (Fig. 6b). The leave-one-out cross validation of the area under the ROC curve (AUC) was 0.959, revealing promising diagnostic potential. The BVS index threshold for a disease diagnosis was set at −0.081, and corresponding sensitivity and specificity were 1.000 and 0.818, respectively.

10.1128/msystems.01357-21.3 FIG S3 LEfSe identifying the biomarkers for BVS diagnosis. Download FIG S3, JPG file, 0.5 MB. Copyright © 2022 Zhao et al. 2022 Zhao et al. https://creativecommons.org/licenses/by/4.0/ This content is distributed under the terms of the Creative Commons Attribution 4.0 International license.

### The culture environment shapes the gut microbiota and correlates with BVS.

To estimate the effects of the living environment on *A. japonicus* with BVS, we used SourceTracker (36) to identify microbe ratios in gut microbiotas sourced from water and sediments in culture ponds (Fig. 6c). We observed almost no microbes of culture water in the healthy *A. japonicus* guts, while significantly higher water-sourced microbes were identified in BVS guts (*P* value < 0.001, Wilcoxon test) (Fig. 6d). In contrast, while more sediment-sourced-microbes were observed in the gut microbiota of *A. japonicus*, levels were similar between BVS and healthy individuals (Fig. 6e). Moreover, principal coordinate analysis (PCoA) and PERMANOVA showed that the overall microbial community composition was significantly different between water or sediment samples from BVS and healthy ponds (Fig. S4). Microbe abundance in water and sediments from different culture ponds was compared at the phylum level to distinguish changes in microbial community composition (Fig. S5). The relative abundance of *Proteobacteria* and *Cyanobacteria* was decreased, and *Bacteroidota* and *Firmicutes* abundance increased in both water and sediment microbial communities from BVS ponds. However, changes in the relative abundance of these phyla in pond water were more obvious than in pond sediments. These results suggested that variations in the water environment could be important factors affecting BVS occurrence in *A. japonicus* aquaculture.

10.1128/msystems.01357-21.4 FIG S4 PCoA for microbial communities in water and sediments from *A. japonicus* culture ponds. Download FIG S4, JPG file, 0.04 MB. Copyright © 2022 Zhao et al. 2022 Zhao et al. https://creativecommons.org/licenses/by/4.0/ This content is distributed under the terms of the Creative Commons Attribution 4.0 International license.

10.1128/msystems.01357-21.5 FIG S5 Differences in dominant phyla in water and sediments from healthy and BVS culture ponds. Download FIG S5, JPG file, 0.2 MB. Copyright © 2022 Zhao et al. 2022 Zhao et al. https://creativecommons.org/licenses/by/4.0/ This content is distributed under the terms of the Creative Commons Attribution 4.0 International license.

### Vitamin B5 supplementation treats BVS in *A. japonicus*.

**FIG 7:**
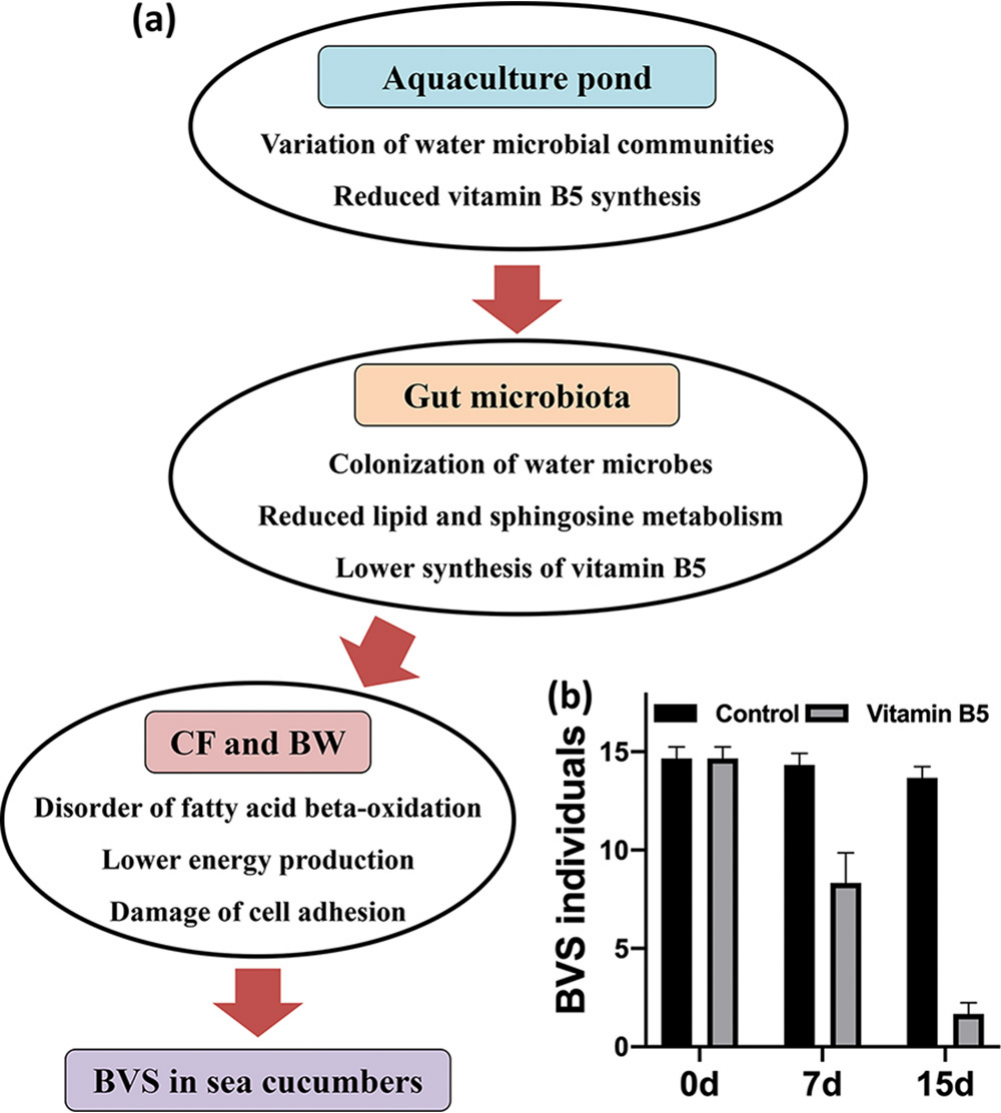
Putative BVS mechanism in *A. japonicus*. (a) Conceptual framework of the putative BVS mechanism in *A. japonicus*. (b) Therapeutic effects of vitamin B5 supplementation on *A. japonicus* with BVS.

We putatively identified vitamin B5 as a key reason for the BVS epidemic in *A. japonicus*. Vitamin B5 is not synthesized by these animals, but it is widely available in their diets (71). Thus, we hypothesized a putative mechanism for BVS occurrence in *A. japonicus* (Fig. 7a). Abnormal water environments in *A. japonicus* aquaculture ponds could alter microbial communities and cause a decline in vitamin B5 synthesis. Consequently, microbial colonization in *A. japonicus* intestines affects lipid and sphingosine metabolism in gut microbiota. A subsequent decrease in secondary metabolites produced by gut microbiota, combined with feed lacking vitamin B5, could disrupt fatty acid β-oxidation. These changes eventually manifest as the BVS phenotype via altered energy metabolism and cell adhesion in the BW of *A. japonicus*.

Based on this putative mechanism, we performed a vitamin B5 supplementation study to observe BVS recovery in *A. japonicus*. After supplementation (0.5 g/L) to animals under laboratory conditions, the BVS incidence decreased from almost 100% to <10% during the trial (Fig. 7b). In contrast, in controls (no vitamin B5 supplementation), no changes in BVS incidences were observed (Fig. 7b). These data not only verified our putative vitamin B5 deficiency mechanism but also indicated the feasibility of vitamin B5 supplementation to treat BVS in *A. japonicus*.

## MATERIALS AND METHODS

### Sample collection.

*A. japonicus* samples were collected from several culture ponds in Jinzhou, China. After collection, animals were immediately dissected and intestine tracts aseptically removed from the body cavity. Intestine contents were then gently squeezed out using phosphate buffer (0.064 M, pH 7.4) and stored in iceboxes. The CF and partial BW of each sample were stored in iceboxes, and the remaining BW was boiled for BVS identification. Finally, we selected 11 BVS and 11 healthy *A. japonicus* animals from three diseased and healthy culture ponds, respectively (Fig. 1c). Surface water (∼5-L) and sediment (∼200-g) samples from each pond were also collected. For proteomics and metabolomics testing, BW and CF samples from the same pond were mixed. All samples were transferred to the laboratory within 24 h under cold conditions and stored at −80°C.

### Histological analysis of BW structures.

BW samples were fixed in 0.1 M phosphate buffer (pH 7.2) plus 10% formaldehyde for at least 24 h. Then, samples were dehydrated in ethyl alcohol, cleared in xylene, transferred to a xylene-wax mixture, and finally embedded in paraﬃn wax. Histological sections were stained in hematoxylin and eosin (H&E) for histomorphological observations under light microscopy (BX53M microscope; Olympus, Germany). BW structures were analyzed using a Zeiss Axioplan 2 high-resolution microscope (Carl Zeiss, Gottingen, Germany), connected to a Leica DC500 color digital camera.

### Label-free proteomics.

Proteins from BW and CF sample were precipitated in trichloroacetic acid for 30 min on ice and centrifuged at 40,000 × *g* for 30 min at 4°C. Protein concentrations were determined using a protein quantification kit (Dingguo Changsheng, Beijing, China), according to the manufacturer's instructions. Then, proteins were excised from the preparative tube and destained in 50 mM NH_4_HCO_3_. Following the addition of 100 mmol/L dl-dithiothreitol at a final concentration of 10 mmol/L, protein fractions were mixed at 56°C for 60 min, diluted 10× in 250 mmol/L iodoacetamide, and stored in the dark for 60 min. Finally, samples were digested using trypsin (substrate to enzyme mass to mass ratio = 50:1) at 37°C for 12 h. Digested peptides were pressure loaded onto a fused silica capillary column, packed with 3 μm Dionex C_18_ material (reverse phase [RP]; Phenomenex) and measured on an Agilent 1100 quaternary high-performance liquid chromatography instrument according to a previous study (53).

Generated tandem mass spectra were searched against the reference *A. japonicus* genome (National Center for Biotechnology Information database accession no. PRJNA413998) using the Mascot search engine (Matrix Science, London, UK; ver. 2.3.02) based on the standard Label-Free Quantification module. Search results were then filtered using a 1% cutoff for false peptide identification rates. Peptides with Z scores of <4 or a delta mass of >5 ppm were rejected. The minimum number of peptides required to identify a protein was one, and proteins with at least two unique peptides were used for abundance quantification. Peptide quantification was based on MS1-level data. For DEP analysis, proteins with missing abundance values in more than one biological replicate of each group were filtered out. In some tissue samples, a protein with two or three abundance values in one group and missing abundance values in all three replicates in another group was identified as unique in this group. The remaining proteins, with fold changes of >1.5 and *P* values of <0.05 (*t* test) between BVS and healthy *A. japonicus*, were considered significant DEPs.

### Nontargeted metabolomics.

For metabolite extraction, 200 mg BW and 100 μL CF were separately added to 300 μL chilled methanol-water (4:1) and vortexed for 3 min. Samples were then incubated at 4°C for 10 min and centrifuged at 13,000 rpm for 10 min. Supernatants were collected and injected onto an Acquity ultrahigh-performance LC (UPLC) HSS T3 column (100 mm by 2.1 mm; 1.8 μm; Waters, UK) to separate metabolites. A Xevo G2 XS QTOF high-resolution tandem mass spectrometer (Waters, UK) was used to detect metabolites eluting from the UPLC column. During analysis, a quality control sample was injected every three samples to monitor repeatability. Runs were performed according to a previous study (72).

MarkerView software (DH Tech. Dev. Pte. Ltd.) was used to extract peak information from raw data to identify metabolite characteristics, including *m/z*, retention times, and ion areas. Then, MetaboAnalyst ver. 3.0 (73) was used to normalize metabolite data for subsequent statistical analyses. The VIP value, Student’s *t* tests, and fold change were used to recognize DAMs at the intersection of the following criteria: (i) a VIP value of ≥1, (ii) a fold change of ≥1.20 or ≤0.83, and (iii) a *P* value of <0.05 (74). By searching for accurate DAM *m/z* values, DAM annotations were performed using the HMDB (75). For more accurate DAM annotations, retention times and ion areas were further screened to compare the structural information of standards.

### High-throughput sequencing of gut microbiota and pond microbiome samples.

Microbial DNA from *A. japonicus* intestinal contents was extracted using the Qiagen DNA stool minikit (Qiagen, CA, USA) following the manufacturer's instructions. Also, environmental DNA from water and sediment samples was extracted using a PowerWater DNA isolation kit (MO BIO, CA, USA) and a FastDNA spin kit for soil (MP Biomedicals, CA, USA), respectively, following the manufacturers’ instructions. We used 1% agarose gel electrophoresis to assess DNA extraction, and then sample concentration and purity were measured using a NanoPhotometer Classic instrument (IMPLEN, Germany).

The V3-V4 region of bacterial 16S rRNA was amplified from DNA using primers 341F and 806R and amplicons sequenced using an Illumina Miseq platform, with a 300-bp paired-end strategy, according to a previous study (3). After quality control, paired-end reads were assembled into tags using FLASH according to overlapped regions (76). Tags were then assigned to amplicon sequence variants (ASVs) using the QIIME (Quantitative Insights Into Microbial Ecology) 2 through the Divisive Amplicon Denoising Algorithm 2 method (77). Representative sequences of each ASV were selected by the default method and assigned to a bacterial taxonomy based on the SILVA release 138 database (78). Singletons (number of a specific ASV = 1) were abandoned to improve data analysis. Finally, a bacterial ASV abundance table was normalized using a standard number of tags according to the sample with the fewest tags (29,451).

### Vitamin B5 supplementation study.

Based on our theoretical BVS mechanism, a vitamin B5 supplementation study was established to verify this mechanism and evaluate its therapeutic effects. In total, 110 *A. japonicus* animals were removed from a culture pond in Jinzhou, China; the BVS incidence rate was almost 100%, as determined by boiling (20 individuals). The remaining animals (90) were randomly distributed across six aquariums (0.7 m length × 0.3 m width) in 0.3 m water, with 15 individuals/aquarium. Next, 0.5 g/L vitamin B5 was added to three aquariums, and the others were used as controls. Environmental conditions were as follows: water temperature, 18°C to 22°C; pH 7.6 to 8.3; 6.0 to 7.5 mg/L dissolved oxygen; and < 0.1 mg/L ammonia nitrogen. After 15 days, animals were collected and boiled to determine BVS ratios.

### Statistical analysis.

PLS-DA, based on protein expression or metabolite abundance in BW and CF samples, was used to evaluate differences in protein and metabolite composition between BVS and healthy individuals. We extracted Kyoto Encyclopedia of Genes and Genomes (KEGG) annotations for proteins and metabolites, and enrichment analyses were conducted in DAVID using these annotations (79). We calculated Bray-Curtis distances for gut microbiotas in all *A. japonicus* individuals based on microbial taxon and function composition, with differences compared using the Wilcoxon rank sum test. Differences in gut microbiota composition structures between BVS and healthy individuals were assessed using PCoA and PERMANOVA, based on Bray-Curtis distances. Gut microbes having significantly different relative abundances between BVS and healthy individuals were identified using the Wilcoxon rank sum test.

LEfSe (69) and SIMPER analyses (8) were used to determine potential BVS biomarkers in the gut microbiota of *A. japonicus*. A BVS index, which reflected differences in the relative abundance of BVS- and health-associated biomarkers, was used to measure gut microbiota shift severity in diseased animals compared with healthy controls. ROC curves with AUC values were generated to see if the BVS index could be used to establish a BVS diagnosis and determine diagnostic thresholds.

PCoA and PERMANOVA were also used to evaluate differences in microbial communities between water and sediments in BVS and healthy animal culture ponds. Bacterial phyla with significantly different relative abundances in water or sediments between BVS and healthy culture ponds were assessed using the Wilcoxon rank sum test. A SourceTracker method (36) was used to generate *A. japonicus* gut microbiota ratios from water and sediments in culture ponds. Differences in ratios of water- and sediment-sourced microbes between BVS and healthy animals were compared using the Wilcoxon rank sum test. The therapeutic effects of vitamin B5 were also assessed using the Wilcoxon test by comparing the recovery of BVS *A. japonicus* animals with controls. All analyses and graphs were generated in R v3.6.3, using the packages vegan, mixOmics, pROC, and ggplot2.

### Data availability.

Sequence files and metadata for microbial samples used in this study have been deposited in NCBI SRA (PRJNA779486).

## References

[1] Toralgranda V, Lovatelli A, Vasconcellos M. 2008. Sea cucumbers. a global review of fisheries and trade. FAO Fish Aquacult Tech Pap 516:1–317.

[2] Yu Z, Zhou Y, Yang H, Hu C. 2014. Bottom culture of the sea cucumber *Apostichopus japonicus*, Selenka (Echinodermata: holothuroidea) in a fish farm, southern China. Aquac Res 45:1434–1441. doi:10.1111/are.12089.

[3] Zhao Z, Jiang J, Pan Y, Dong Y, Chen Z, Zhang G, Gao S, Sun H, Guan X, Wang B, Xiao Y, Zhou Z. 2020. Temporal dynamics of bacterial communities in the water and sediments of sea cucumber (*Apostichopus japonicus*) culture ponds. Aquaculture 528:735498. doi:10.1016/j.aquaculture.2020.735498.

[4] Wang YG, Zhang CY, Rong XJ, Chen JJ, Shi YC. 2005. Diseases of cultured sea cucumber, *Apostichopus japonicus*, in China. FAO Fish Tech Pap 46:297–310.

[5] Dong Y, Deng H, Sui X, Song L. 2005. Ulcer disease of farmed sea cucumber (*Apostichopus japonicus*. Fish Sci 24:4–6. (In Chinese).

[6] Deng H, Zhou Z, Wang N, Liu C. 2008. The syndrome of sea cucumber (*Apostichopus japonicus*) infected by virus and bacteria. Virol Sin 23:63–67. doi:10.1007/s12250-008-2863-9.

[7] Yang A, Zhou Z, Pan Y, Jiang J, Dong Y, Guan X, Sun H, Gao S, Chen Z. 2016. RNA sequencing analysis to capture the transcriptome landscape during skin ulceration syndrome progression in sea cucumber *Apostichopus japonicus*. BMC Genomics 17:459. doi:10.1186/s12864-016-2810-3. 27296384 PMC4906609

[8] Zhang Z, Xing R, Lv Z, Shao Y, Zhang W, Zhao X, Li C. 2018. Analysis of gut microbiota revealed *Lactococcus garviaeae* could be an indicative of skin ulceration syndrome in farmed sea cucumber *Apostichopus japonicus*. Fish Shellfish Immunol 80:148–154. doi:10.1016/j.fsi.2018.06.001. 29864588

[9] Song X, Feng Z, Zhang Y, Zhu W. 2019. Regulation of dietary astragalus polysaccharide (APS) supplementation on the non-specific immune response and intestinal microbiota of sea cucumber *Apostichopus japonicus*. Fish Shellfish Immunol 94:517–524. doi:10.1016/j.fsi.2019.09.049. 31542494

[10] Zhao Z, Zhou Z, Dong Y, Pan Y, Jiang J, Jiang Y, Chen Z, Gao S, Wang B, Jiang B. 2021. Association of intestinal fungal communities with the body vesicular syndrome: an emerging disease of sea cucumber (*Apostichopus japonicus*). Aquaculture 530:735758. doi:10.1016/j.aquaculture.2020.735758.

[11] Bureau of Fisheries. 2018. Ministry of Agriculture and Rural Affairs of the People’s Republic of China, National Fisheries Technology Extension Center, China Society of Fisheries, China fisheries statistics yearbook of 2018. China Agricultural Press, Beijing, China.

[12] Peng XX. 2013. Proteomics and its applications to aquaculture in China: infection, immunity, and interaction of aquaculture hosts with pathogens. Dev Comp Immunol 39:63–71. doi:10.1016/j.dci.2012.03.017. 22484215

[13] Alfaro AC, Young T. 2018. Showcasing metabolomic applications in aquaculture: a review. Rev Aquacult 10:135–152. doi:10.1111/raq.12152.

[14] Ahmed F, Kumar G, Soliman FM, Adly MA, Soliman HA, El-Matbouli M, Saleh M. 2019. Proteomics for understanding pathogenesis, immune modulation and host pathogen interactions in aquaculture. Comp Biochem Phys D 32:100625. doi:10.1016/j.cbd.2019.100625. 31639560

[15] Zhao Z, Jiang J, Pan Y, Sun H, Guan X, Gao S, Chen Z, Dong Y, Zhou Z. 2018. Proteomic analysis reveals the important roles of alpha-5-collagen and ATP5β during skin ulceration syndrome progression of sea cucumber *Apostichopus japonicus*. J Proteomics 175:136–143. doi:10.1016/j.jprot.2018.01.001. 29325989

[16] Shao Y, Li C, Ou C, Zhang P, Lu Y, Su X, Li Y, Li T. 2013. Divergent metabolic responses of *Apostichopus japonicus* suffered from skin ulceration syndrome and pathogen challenge. J Agric Food Chem 61:10766–10771. doi:10.1021/jf4038776. 24127639

[17] Berry D, Schwab C, Milinovich G, Reichert J, Mahfoudh KB, Decker T, Engel M, Hai B, Hainzl E, Heider S, Kenner L, Muller M, Rauch I, Strobl B, Wagner M, Schleper C, Urich T, Loy A. 2012. Phylotype-level 16S rRNA analysis reveals new bacterial indicators of health state in acute murine colitis. ISME J 6:2091–2106. doi:10.1038/ismej.2012.39. 22572638 PMC3475367

[18] Clemente JC, Ursell LK, Parfrey LW, Knight R. 2012. The impact of the gut microbiota on human health: an integrative view. Cell 148:1258–1270. doi:10.1016/j.cell.2012.01.035. 22424233 PMC5050011

[19] Subramanian S, Huq S, Yatsunenko T, Haque R, Mahfuz M, Alam MA, Benezra A, DeStefano J, Meier MF, Muegge BD, Barratt MJ, van Arendonk LG, Zhang Q, Province MA, Petri WA, Jr, Ahmed T, Gordon JI. 2014. Persistent gut microbiota immaturity in malnourished Bangladeshi children. Nature 510:417–421. doi:10.1038/nature13421. 24896187 PMC4189846

[20] Franzosa EA, Sirota-Madi A, Avila-Pacheco J, Fornelos N, Haiser HJ, Reinker S, Vatanen T, Hall AB, Mallick H, McIver LJ, Sauk JS, Wilson RG, Stevens BW, Scott JM, Pierce K, Deik AA, Bullock K, Imhann F, Porter JA, Zhernakova A, Fu J, Weersma RK, Wijmenga C, Clish CB, Vlamakis H, Huttenhower C, Xavier RJ. 2019. Gut microbiome structure and metabolic activity in inflammatory bowel disease. Nat Microbiol 4:293–305. doi:10.1038/s41564-018-0306-4. 30531976 PMC6342642

[21] Liu R, Zhang C, Shi Y, Zhang F, Li L, Wang X, Ling Y, Fu H, Dong W, Shen J, Reeves A, Greenberg AS, Zhao L, Peng Y, Ding X. 2017. Dysbiosis of gut microbiota associated with clinical parameters in polycystic ovary syndrome. Front Microbiol 8:324. doi:10.3389/fmicb.2017.00324. 28293234 PMC5328957

[22] Liu R, Hong J, Xu X, Feng Q, Zhang D, Gu Y, Shi J, Zhao S, Liu W, Wang X, Xia H, Liu Z, Cui B, Liang P, Xi L, Jin J, Ying X, Wang X, Zhao X, Li W, Jia H, Lan Z, Li F, Wang R, Sun Y, Yang M, Shen Y, Jie Z, Li J, Chen X, Zhong H, Xie H, Zhang Y, Gu W, Deng X, Shen B, Xu X, Yang H, Xu G, Bi Y, Lai S, Wang J, Qi L, Madsen L, Wang J, Ning G, Kristiansen K, Wang W. 2017. Gut microbiome and serum metabolome alterations in obesity and after weight-loss intervention. Nat Med 23:859–868. doi:10.1038/nm.4358. 28628112

[23] Xiong JB, Nie L, Chen J. 2019. Current understanding on the roles of gut microbiota in fish disease and immunity. Zoo Res 40:70–76. doi:10.24272/j.issn.2095-8137.2018.069. PMC6378566 29976843

[24] Dai W, Sheng Z, Chen J, Xiong J. 2020. Shrimp disease progression increases the gut bacterial network complexity and abundances of keystone taxa. Aquaculture 517:734802. doi:10.1016/j.aquaculture.2019.734802.

[25] Deng H, He C, Zhou Z, Liu C, Tan K, Wang N, Jiang B, Gao X, Liu W. 2009. Isolation and pathogenicity of pathogens from skin ulceration disease and viscera ejection syndrome of the sea cucumber *Apostichopus japonicus*. Aquaculture 287:18–27. doi:10.1016/j.aquaculture.2008.10.015.

[26] Boulangé CL, Neves AL, Chilloux J, Nicholson JK, Dumas ME. 2016. Impact of the gut microbiota on inflammation, obesity, and metabolic disease. Genome Med 8:42. doi:10.1186/s13073-016-0303-2. 27098727 PMC4839080

[27] Tasnim N, Abulizi N, Pither J, Hart MM, Gibson DL. 2017. Linking the gut microbial ecosystem with the environment: does gut health depend on where we live? Front Microbiol 8:1935. doi:10.3389/fmicb.2017.01935. 29056933 PMC5635058

[28] Sun J, Liao XP, D’Souza AW, Boolchandani M, Li SH, Cheng K, Martinez JL, Li L, Feng YJ, Fang LX, Huang T, Xia J, Yu Y, Zhou YF, Sun YX, Deng XB, Zeng ZL, Jiang HX, Fang BH, Tang YZ, Lian XL, Zhang RM, Fang ZW, Yan QL, Dantas G, Liu YH. 2020. Environmental remodeling of human gut microbiota and antibiotic resistome in livestock farms. Nat Commun 11:1427. doi:10.1038/s41467-020-15222-y. 32188862 PMC7080799

[29] Hauksson E. 1979. Feeding biology of *Stichopus tremulus*, a deposit feeding holothurian. Sarsia 64:155–160. doi:10.1080/00364827.1979.10411376.

[30] Gao F, Qiang XU, Yang HS. 2010. Seasonal variations of food sources in *Apostichopus japonicus* indicated by fatty acid biomarkers analysis. J Fish China 34:760–767. (In Chinese.) doi:10.3724/SP.J.1231.2010.06768.

[31] Yang H, Yuan X, Zhou Y, Mao Y, Zhang T, Liu Y. 2005. Effects of body size and water temperature on food consumption and growth in the sea cucumber *Apostichopus japonicus* (Selenka) with special reference to aestivation. Aquaculture Res 36:1085–1092. doi:10.1111/j.1365-2109.2005.01325.x.

[32] Yang H, Hamel JF, Mercier A (ed). 2015. The sea cucumber Apostichopus japonicus: history, biology and aquaculture. Academic Press, New York, NY.

[33] Bai MM. 1971. Regeneration in the holothurian, *holothuria scabra jager*. Indian J Exp Biol 9:467–471. 5147174

[34] Li Y, Wang R, Xu X, Wang J, Bao L, Thimmappa R, Ding J, Jiang J, Zhang L, Li T, Lv J, Mu C, Hu X, Zhang L, Liu J, Li Y, Yao L, Jiao W, Wang Y, Lian S, Zhao Z, Zhan Y, Huang X, Liao H, Wang J, Sun H, Mi X, Xia Y, Xing Q, Liu W, Osbourn A, Zhou Z, Chang Y, Bao Z, Wang S. 2018. Sea cucumber genome provides insights into saponin biosynthesis and aestivation regulation. Cell Discov 4:29. doi:10.1038/s41421-018-0030-5. 29951224 PMC6018497

[35] Shukalyuk AI, Dolmatov IY. 2001. Regeneration of the digestive tube in the holothurian *Apostichopus japonicusafter* evisceration. Russ J Mar Biol 27:168–173. doi:10.1023/A:1016717502616.

[36] Knights D, Kuczynski J, Charlson ES, Zaneveld J, Mozer MC, Collman RG, Bushman FD, Knight R, Kelley ST. 2011. Bayesian community-wide culture-independent microbial source tracking. Nat Methods 8:761–763. doi:10.1038/nmeth.1650. 21765408 PMC3791591

[37] Jones G, Prosser DE, Kaufmann M. 2014. Cytochrome P450-mediated metabolism of vitamin D. J Lipid Res 55:13–31. doi:10.1194/jlr.R031534. 23564710 PMC3927478

[38] Tanaka Y, Kasahara K, Izawa M, Ochi K. 2017. Applicability of ribosome engineering to vitamin B12 production by *Propionibacterium Shermanii*. Biosci Biotech Bioch 81:1636–1641. doi:10.1080/09168451.2017.1329619. 28532245

[39] Amrein K, Oudemans-van Straaten HM, Berger MM. 2018. Vitamin therapy in critically ill patients: focus on thiamine, vitamin C, and vitamin D. Intensive Care Med 44:1940–1944. doi:10.1007/s00134-018-5107-y. 29520660 PMC6244527

[40] Xu C, Ng DT. 2015. Glycosylation-directed quality control of protein folding. Nat Rev Mol Cell Biol 16:742–752. doi:10.1038/nrm4073. 26465718

[41] Xu G, Goonatilleke E, Wongkham S, Lebrilla CB. 2020. Deep structural analysis and quantitation of O-linked glycans on cell membrane reveal high abundances and distinct glycomic profiles associated with cell type and stages of differentiation. Anal Chem 92:3758–3768. doi:10.1021/acs.analchem.9b05103. 32039582 PMC11758683

[42] Misevic G, Garbarino E. 2021. Glycan-to-glycan binding: molecular recognition through polyvalent interactions mediates specific cell adhesion. Molecules 26:397. doi:10.3390/molecules26020397. PMC7828597 33451117

[43] Wang CC, Chen JR, Tseng YC, Hsu CH, Hung YF, Chen SW, Chen CM, Khoo KH, Cheng TJ, Cheng YSE, Jan JT, Wu CY, Ma C, Wong CH. 2009. Glycans on influenza hemagglutinin affect receptor binding and immune response. Proc Natl Acad Sci USA 106:18137–18142. doi:10.1073/pnas.0909696106. 19822741 PMC2775302

[44] Pickart CM, Eddins MJ. 2004. Ubiquitin: structures, functions, mechanisms. BBA Mol Cell Res 1695:55–72. doi:10.1016/j.bbamcr.2004.09.019. 15571809

[45] Bertoli E, Parenti-Castelli G, Sechi AM, Trigari G, Lenaz G. 1978. A requirement for ubiquinone in ATPase activity and oxidative phosphorylation. Biochem Bioph Res Co 85:1–6. doi:10.1016/S0006-291X(78)80002-3. 154324

[46] Ernster L, Dallner G. 1995. Biochemical, physiological and medical aspects of ubiquinone function. BBA-Mol Basis Dis 1271:195–204. doi:10.1016/0925-4439(95)00028-3. 7599208

[47] Chen CS, Alonso JL, Ostuni E, Whitesides GM, Ingber DE. 2003. Cell shape provides global control of focal adhesion assembly. Biochem Bioph Res Co 307:355–361. doi:10.1016/S0006-291X(03)01165-3. 12859964

[48] Pollard TD, Cooper JA. 1986. Actin and actin-binding proteins. A critical evaluation of mechanisms and functions. Annu Rev Biochem 55:987–1035. doi:10.1146/annurev.bi.55.070186.005011. 3527055

[49] Critchley DR. 2004. Cytoskeletal proteins talin and vinculin in integrin-mediated adhesion. Biochem Soc Trans 32:831–836. doi:10.1042/BST0320831. 15494027

[50] Emsley J, Knight CG, Farndale RW, Barnes MJ, Liddington RC. 2000. Structural basis of collagen recognition by integrin α2β1. Cell 101:47–56. doi:10.1016/S0092-8674(00)80622-4. 10778855

[51] Blanchard A, Ohanian V, Critchley D. 1989. The structure and function of α-actinin. J Muscle Res Cell Motil 10:280–289. doi:10.1007/BF01758424. 2671039

[52] Gheldof A, Berx G. 2013. Cadherins and epithelial-to-mesenchymal transition. Prog Mol Biol Transl Sci 116:317–336. doi:10.1016/B978-0-12-394311-8.00014-5. 23481201

[53] Jiang J, Zhao Z, Pan Y, Dong Y, Gao S, Jiang B, Xiao Y, Jiang P, Zhang G, Wang X, Zhou Z. 2020. Proteomics reveals the gender differences in humoral immunity and physiological characteristics associated with reproduction in the sea cucumber *Apostichopus japonicus*. J Proteomics 217:103687. doi:10.1016/j.jprot.2020.103687. 32061807

[54] Rodrigues RG, Guo N, Zhou L, Sipes JM, Williams SB, Templeton NS, Gralnick HR, Roberts DD. 2001. Conformational regulation of the fibronectin binding and α3β1 integrin-mediated adhesive activities of thrombospondin-1. J Biol Chem 276:27913–27922. doi:10.1074/jbc.M009518200. 11358957

[55] Purslow PP. 2020. The structure and role of intramuscular connective tissue in muscle function. Front Physiol 11:495. doi:10.3389/fphys.2020.00495. 32508678 PMC7248366

[56] Pollard TD. 2007. Regulation of actin filament assembly by Arp2/3 complex and formins. Annu Rev Biophys Biomol Struct 36:451–477. doi:10.1146/annurev.biophys.35.040405.101936. 17477841

[57] Trotter JA. 2002. Structure–function considerations of muscle–tendon junctions. Comp Biochem Phys A 133:1127–1133. doi:10.1016/S1095-6433(02)00213-1. 12485696

[58] Dashty M, Motazacker MM, Levels J, de Vries M, Mahmoudi M, Peppelenbosch MP, Rezaee F. 2014. Proteome of human plasma very low-density lipoprotein and low-density lipoprotein exhibits a link with coagulation and lipid metabolism. Thromb Haemost 111:518–530. doi:10.1160/TH13-02-0178. 24500811

[59] Knottnerus SJ, Bleeker JC, Wüst RC, Ferdinandusse S, Ijlst L, Wijburg FA, Wanders RJA, Visser G, Houtkooper RH. 2018. Disorders of mitochondrial long-chain fatty acid oxidation and the carnitine shuttle. Rev Endocr Metab Disord 19:93–106. doi:10.1007/s11154-018-9448-1. 29926323 PMC6208583

[60] Le Borgne F, Mohamed AB, Logerot M, Garnier E, Demarquoy J. 2011. Changes in carnitine octanoyltransferase activity induce alteration in fatty acid metabolism. Biochem Biophys Res Commun 409:699–704. doi:10.1016/j.bbrc.2011.05.068. 21619872

[61] Vishwanath VA. 2016. Fatty acid beta-oxidation disorders: a brief review. Ann Neurosci 23:51–55. doi:10.1159/000443556. 27536022 PMC4934411

[62] Leonardi R, Jackowski S. 2007. Biosynthesis of pantothenic acid and coenzyme A. EcoSal Plus 2 doi:10.1128/ecosalplus.3.6.3.4. PMC4950986 26443589

[63] Wang L, Ravichandran V, Yin Y, Yin J, Zhang Y. 2019. Natural products from mammalian gut microbiota. Trends Biotechnol 37:492–504. doi:10.1016/j.tibtech.2018.10.003. 30392727

[64] Brown EM, Ke X, Hitchcock D, Jeanfavre S, Avila-Pacheco J, Nakata T, Arthur TD, Fornelos N, Heim C, Franzosa EA, Watson N, Huttenhower C, Haiser HJ, Dillow G, Graham DB, Finlay BB, Kostic AD, Porter JA, Vlamakis H, Clish CB, Xavier RJ. 2019. Bacteroides-derived sphingolipids are critical for maintaining intestinal homeostasis and symbiosis. Cell Host Microbe 25:668–680. doi:10.1016/j.chom.2019.04.002. 31071294 PMC6544385

[65] Kolesnick RN. 1991. Sphingomyelin and derivatives as cellular signals. Prog Lipid Res 30:1–38. doi:10.1016/0163-7827(91)90005-p. 1771169

[66] Houten SM, Watanabe M, Auwerx J. 2006. Endocrine functions of bile acids. EMBO J 25:1419–1425. doi:10.1038/sj.emboj.7601049. 16541101 PMC1440314

[67] Wahlström A, Sayin SI, Marschall HU, Bäckhed F. 2016. Intestinal crosstalk between bile acids and microbiota and its impact on host metabolism. Cell Metab 24:41–50. doi:10.1016/j.cmet.2016.05.005. 27320064

[68] Xiong J, Zhu J, Dai W, Dong C, Qiu Q, Li C. 2017. Integrating gut microbiota immaturity and disease‐discriminatory taxa to diagnose the initiation and severity of shrimp disease. Environ Microbiol 19:1490–1501. doi:10.1111/1462-2920.13701. 28205371

[69] Segata N, Izard J, Waldron L, Gevers D, Miropolsky L, Garrett WS, Huttenhower C. 2011. Metagenomic biomarker discovery and explanation. Genome Biol 12:R60. doi:10.1186/gb-2011-12-6-r60. 21702898 PMC3218848

[70] Zhao L, Zhang F, Ding X, Wu G, Lam YY, Wang X, Fu H, Xue X, Lu C, Ma J, Yu L, Xu CM, Ren Z, Xu Y, Xu S, Shen H, Zhu X, Shi Y, Shen Q, Dong W, Liu R, Ling Y, Zeng Y, Wang X, Zhang QP, Wang J, Wang L, Wu Y, Zeng B, Wei H, Zhang M, Peng Y, Zhang C. 2018. Gut bacteria selectively promoted by dietary fibers alleviate type 2 diabetes. Science 359:1151–1156. doi:10.1126/science.aao5774. 29590046

[71] Sweetman L. 2004. Pantothenic acid, p 517–525. *In* Coates PM, Blackman MR, Cragg GM, Levine M, Moss J, White JD (ed). Encyclopedia of dietary supplements. CRC Press, Boca Raton, FL.

[72] Jiang J, Zhao Z, Gao S, Chen Z, Dong Y, He P, Wang B, Pan Y, Wang X, Guan X, Wang C, Lin S, Sun H, Zhou Z. 2021. Divergent metabolic responses to sex and reproduction in the sea cucumber *Apostichopus japonicus*. Comp Biochem Physiol Part D Genomics Proteomics 39:100845. doi:10.1016/j.cbd.2021.100845. 33971398

[73] Pang Z, Chong J, Li S, Xia J. 2020. MetaboAnalystR 3.0: toward an optimized workflow for global metabolomics. Metabolites 10:186. doi:10.3390/metabo10050186. PMC7281575 32392884

[74] Yin P, Jia A, Heimann K, Zhang M, Liu X, Zhang W, Liu C. 2020. Hot water pretreatment-induced significant metabolite changes in the sea cucumber *Apostichopus japonicus*. Food Chem 314:126211. doi:10.1016/j.foodchem.2020.126211. 31982856

[75] Wishart DS, Tzur D, Knox C, Eisner R, Guo AC, Young N, Cheng D, Jewell K, Arndt D, Sawhney S, Fung C, Nikolai L, Lewis M, Coutouly M-A, Forsythe I, Tang P, Shrivastava S, Jeroncic K, Stothard P, Amegbey G, Block D, Hau DD, Wagner J, Miniaci J, Clements M, Gebremedhin M, Guo N, Zhang Y, Duggan GE, Macinnis GD, Weljie AM, Dowlatabadi R, Bamforth F, Clive D, Greiner R, Li L, Marrie T, Sykes BD, Vogel HJ, Querengesser L. 2007. HMDB: the human metabolome database. Nucleic Acids Res 35:D521–D526. doi:10.1093/nar/gkl923. 17202168 PMC1899095

[76] Magoc T, Salzberg SL. 2011. FLASH: fast length adjustment of short reads to improve genome assemblies. Bioinformatics 27:2957–2963. doi:10.1093/bioinformatics/btr507. 21903629 PMC3198573

[77] Bokulich NA, Kaehler BD, Rideout JR, Dillon M, Bolyen E, Knight R, Huttley GA, Caporaso JG. 2018. Optimizing taxonomic classification of marker-gene amplicon sequences with QIIME 2’s q2-feature-classifier plugin. Microbiome 6:90. doi:10.1186/s40168-018-0470-z. 29773078 PMC5956843

[78] Yilmaz P, Parfrey LW, Yarza P, Gerken J, Pruesse E, Quast C, Schweer T, Peplies J, Ludwig W, Glöckner FO. 2014. The SILVA and “all-species living tree project (LTP)” taxonomic frameworks. Nucleic Acids Res 42:D643–D648. doi:10.1093/nar/gkt1209. 24293649 PMC3965112

[79] Huang D, Sherman B, Lempicki R. 2009. Systematic and integrative analysis of large gene lists using DAVID bioinformatics resources. Nat Protoc 4:44–57. doi:10.1038/nprot.2008.211. 19131956

