# Supplementary figures and images for: Sea Cucumber Body Vesicular Syndrome Is Driven by the Pond Water Microbiome via an Altered Gut Microbiota
Source: mSystems. 2022 Apr 14;7(3):e01357-21. doi: 10.1128/msystems.01357-21 (PMC9239130; doi:10.1128/msystems.01357-21)

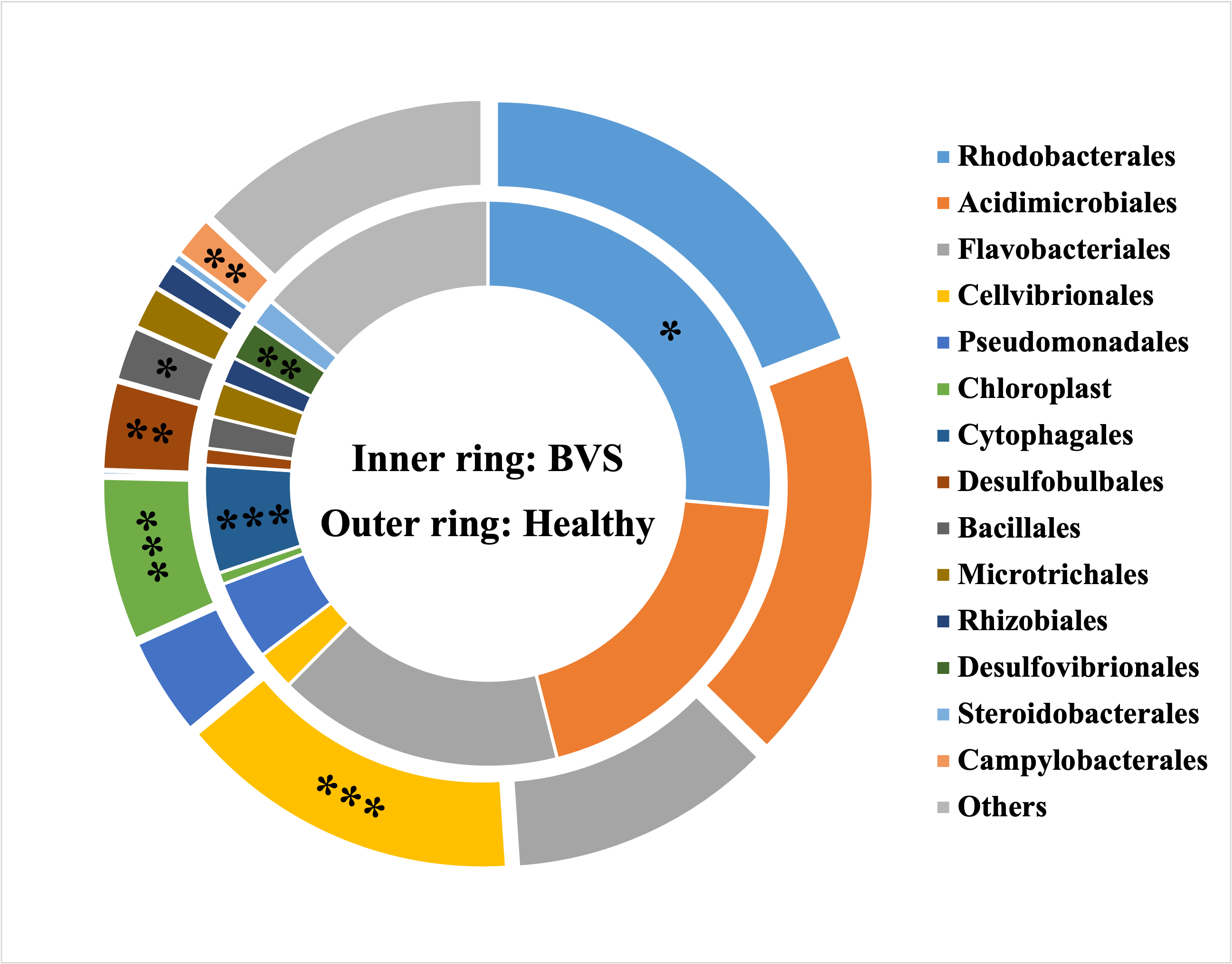

Supplement: FIG S1 [file msystems.01357-21-sf001.jpg]

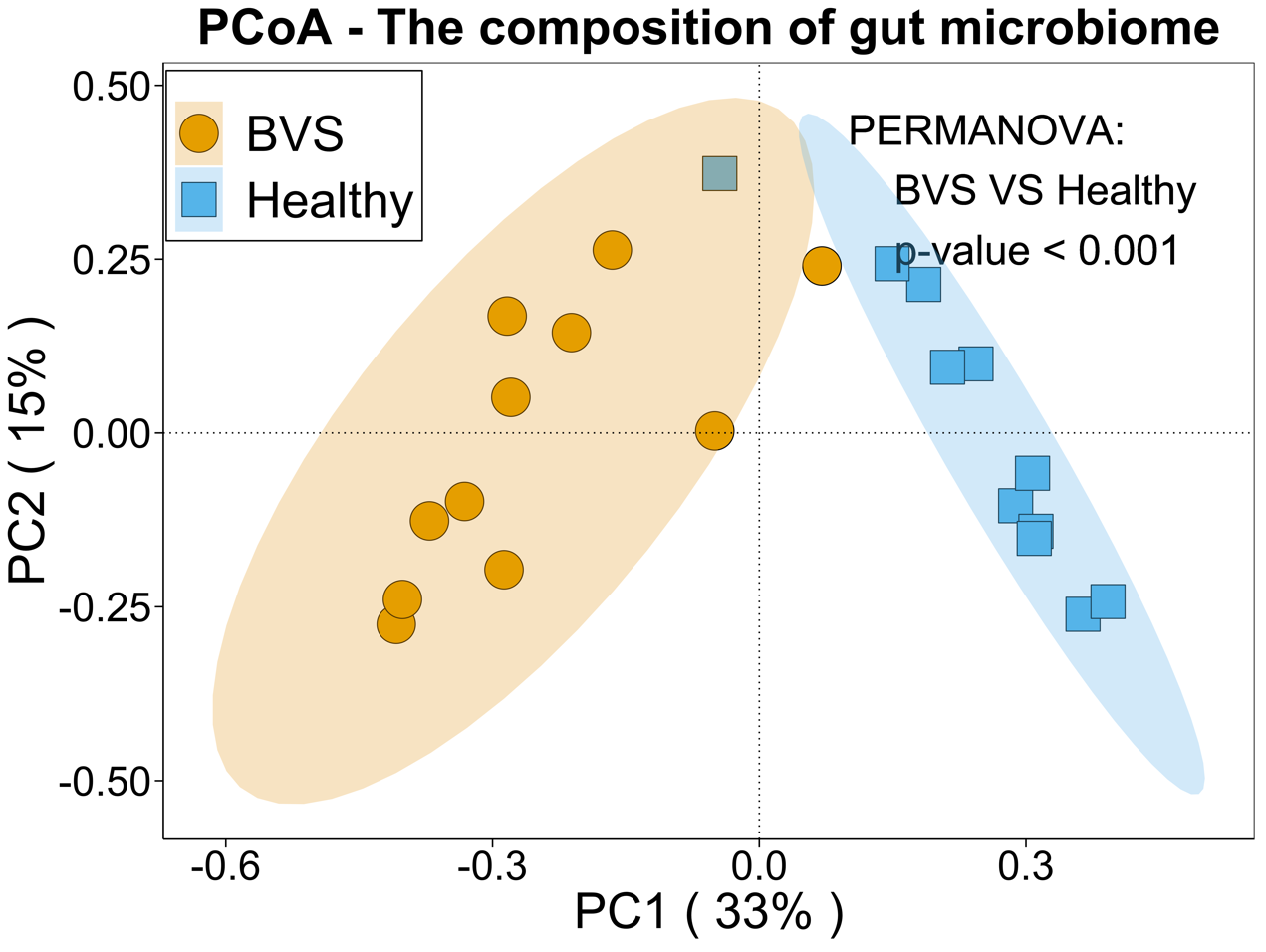

Supplement: FIG S2 [file msystems.01357-21-sf002.jpg]

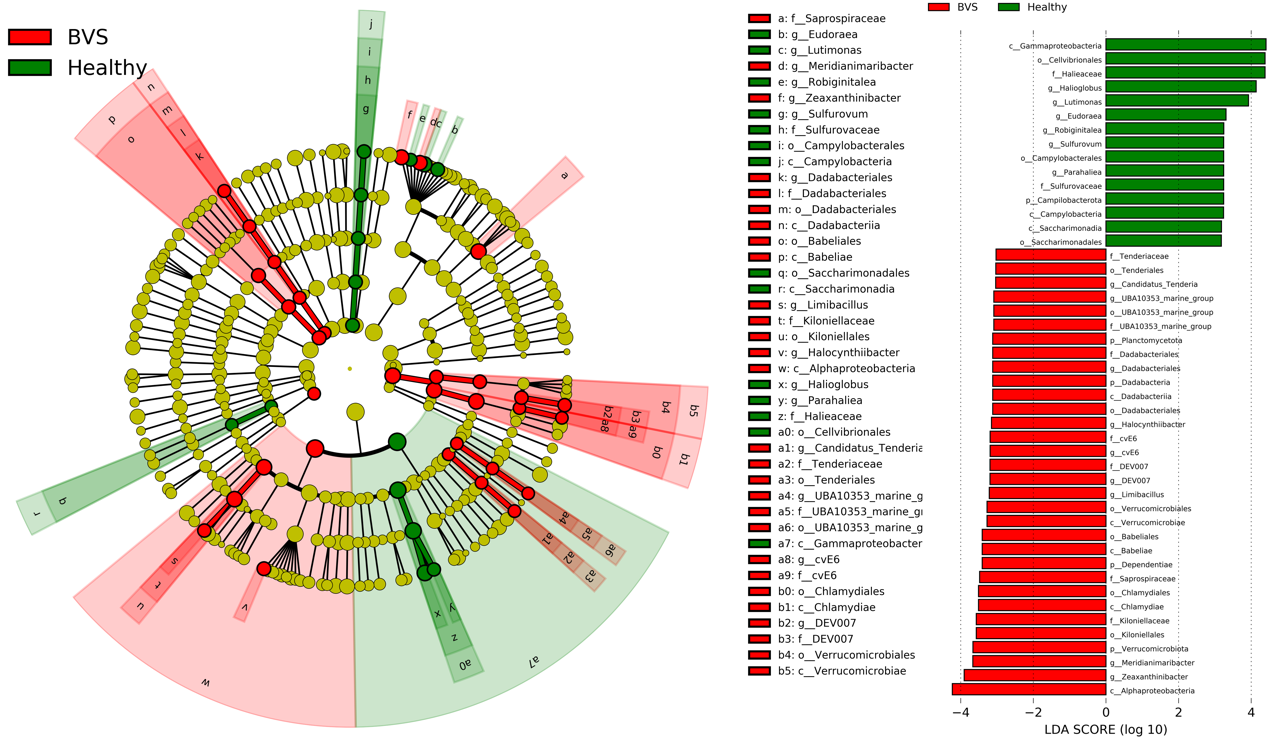

Supplement: FIG S3 [file msystems.01357-21-sf003.jpg]

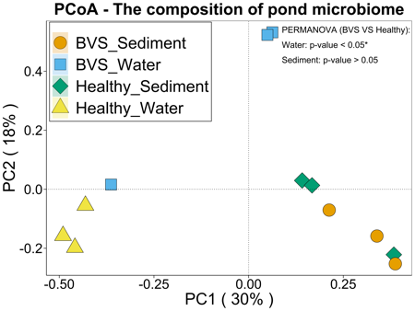

Supplement: FIG S4 [file msystems.01357-21-sf004.jpg]

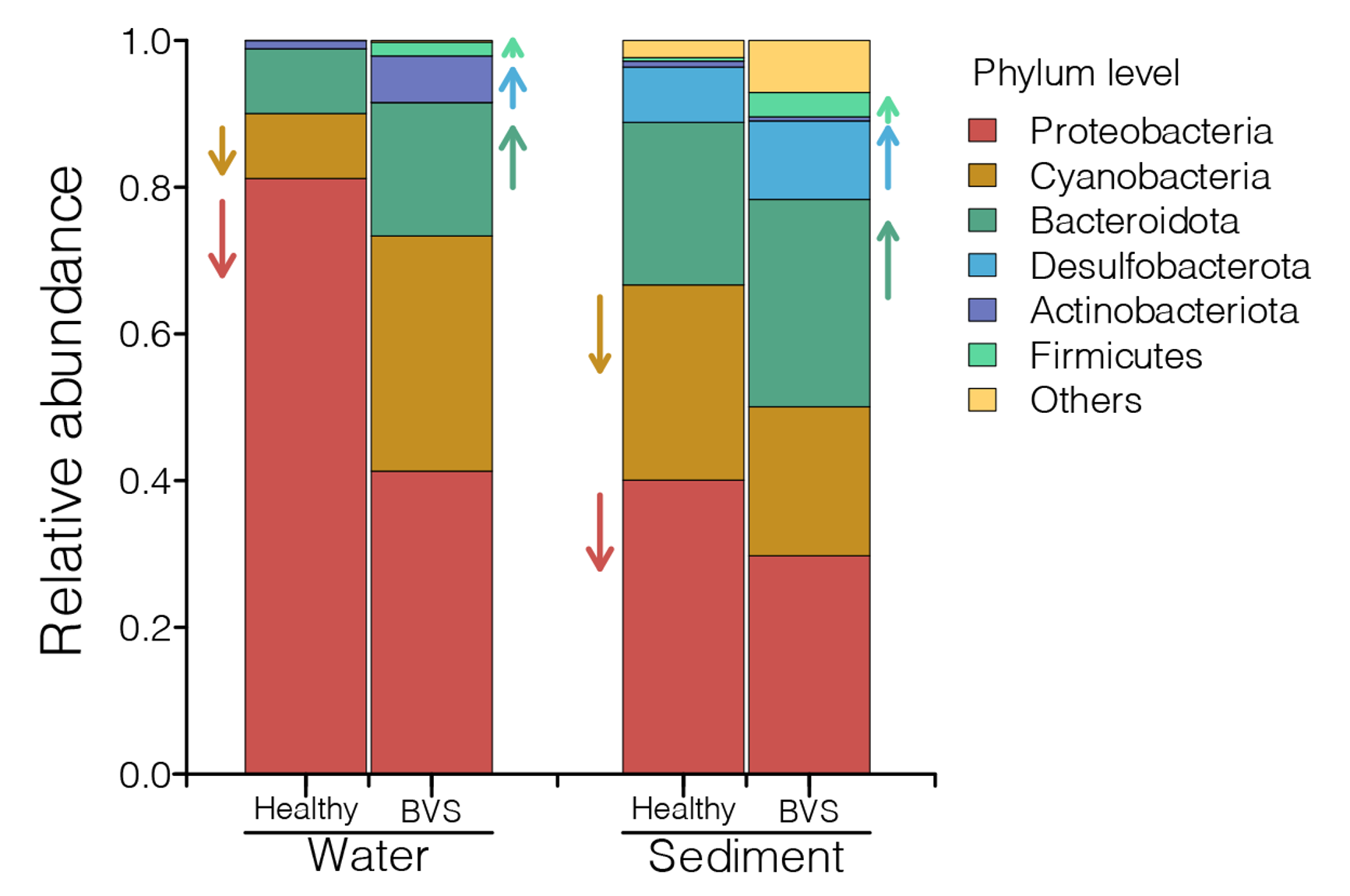

Supplement: FIG S5 [file msystems.01357-21-sf005.jpg]
